# Effect of Polishing on Lead and Cadmium Bioavailability in Rice and Its Health Implications

**DOI:** 10.3390/foods11172718

**Published:** 2022-09-05

**Authors:** Xianghua Chen, Fangman Chen, Shuo Sun, Yingwen Li, Yongxing Li, Hui Mo, Zhian Li, Ping Zhuang

**Affiliations:** 1 South China Botanical Garden, Chinese Academy of Sciences, Guangzhou 510650, China; 2 Southern Marine Science and Engineering Guangdong Laboratory (Guangzhou), Guangzhou 511458, China; 3 University of Chinese Academy of Sciences, Beijing 100049, China

**Keywords:** rice polishing, lead, cadmium, bioavailability, risk assessment

## Abstract

Rice polishing is an important approach to reducing the concentrations of heavy metals in rice, but knowledge of its effect on the Pb and Cd bioavailability in produced rice and the related health risk remains limited. In this study, the effects of rice polishing on the bioaccessibility (BAC) and bioavailability (RBA) of Pb and Cd in rice are assessed using an in vitro method and an in vivo mouse bioassay. The Pb removal rate in brown rice (40%), lightly processed brown rice (62%), germinated rice (74%), and polished rice (79%) gradually enhanced with an increase in the polishing degree, while Cd was difficult to remove by polishing. The Pb and Cd BAC in germinated rice was the highest, while that in brown rice was the lowest. The polished rice Pb and Cd RBA in the liver and kidneys were significantly higher than those in the brown rice group. The Pb RBA in the livers and kidneys in the polished rice group was 26.6% ± 1.68% and 65.3% ± 0.83%, respectively, which was 1.6- and 2.6-times higher than that in the brown rice group, respectively. The Cd RBA values in both the livers and kidneys of the polished rice group were 1.3-times higher than those in the brown rice group. Although polishing reduced the total Pb in the polished rice, it was not enough to offset the increase in bioavailability, and its consumption risk was not weakened. This study highlighted the value of the oral-bioavailability-corrected health risk assessment for assessing the influence of rice polishing on Pb and Cd exposure via rice consumption.

## 1. Introduction

Rice is the staple food for over half the world’s population. China and India alone account for approximately 50% of the rice grown and consumed [1]. Among the exposure pathways, the consumption of rice contaminated with lead (Pb) and cadmium (Cd) comprises a sizable proportion of the overall daily Pb and Cd intake [2,3]. The maximum permissible limits for Pb (0.2, 0.2, 0.2 mg kg^−1^) and Cd (0.2, 0.1, 0.4 mg kg^−^^1^) in rice grain were established by China, the EU, and the WHO, respectively. Lead and Cd are listed as the second- and seventh-most-hazardous substances by the Agency for Toxic Substances and Disease Registry [4], respectively. An overdose of Pb exposure will cause permanent damage to the human nervous system, immune system, and reproductive system and even agglomerate in human bones for decades. Cd is a non-essential heavy metal in the human body, and poisoning primarily manifests itself in bone mineralization, kidney damage, cerebral infarction, and induced cancer [5]. Due to long-term exposure to heavy metal pollution, the cancer incidence rate in Shangba village near the Dabaoshan Mine in Shaoguan, Guangdong Province, China, is greater than nine-times the national average, and the cancer fatality rate is as high as 56% [6]. Therefore, there is a pressing need to develop some strategies to minimize dietary exposure to Pb and Cd from rice to enhance public health.

In human health risk assessments, bioaccessibility and bioavailability have been widely used instead of the total amount to evaluate the risk to human health caused by heavy metals [7]. Bioaccessibility refers to the fraction of a contaminant mobilized from the substance (such as food and drug) by in vitro methods, and the contaminant has the potential to be absorbed during gastrointestinal digestion [8]. The simulated digestive organs, the solid–liquid ratio, the composition of the simulated fluid, pH, and digestion time are factors that influence gastrointestinal digestion in vitro. Bioavailability is defined as the percentage of a contaminant that actually enters the systemic circulation causing either positive or negative effects [7]. Animal assays (e.g., mice, swine, and rabbits) have been applied to determine the oral bioavailability of pollutants in contaminated soils and vegetables [9,10,11]. The metabolic kinetics of mice are similar to that of humans, and mice have the advantages of lower cost and ease of operation and are suitable for breeding in laboratories [12]. The bioavailability of Pb was determined under steady-state exposure in which soil was made into animal feed and fed to mice within 10 days [13].

Currently, treatment technologies for polluted farmlands remain far from being practical and cannot be popularized and applied at a large scale. It is difficult to solve the problem of excessive heavy metals in agricultural products in a short time. Therefore, modulating the bioavailability and absorption of heavy metals during rice processing and consumption might be a new method that can alleviate the toxic effects and hazards of heavy metals. In recent years, some researchers have sought countermeasures to reduce heavy metal exposure in the human body, including rice polishing, component separation, solvent extraction, microbial fermentation, and dietary strategies. As a rice production process, rice polishing refers to the process of turning rice grain into edible rice. This process primarily removes the husks from the rice grain. The concentrations of heavy metals in rice are influenced by the processing precision. Previous research has shown that the reduction rates of heavy metals varied after polishing rice samples using different processing precisions [14,15,16]. Since the heavy metal distribution in rice is different, the removal rate of heavy metals from different rice varieties may be different through rice polishing. In addition, nutrients in rice or the combination morphology of heavy metals may vary in rice with different processing precisions, and this can affect the bioavailability and consumption risk of heavy metals. However, most of these early studies focused on reducing the concentrations of heavy metals using rice polishing, but no study has considered the rice consumption risk after polishing.

The objective of this study was to determine the influence of rice polishing on the concentrations and relative bioavailability (RBA) of Pb and Cd, which were studied using an in vitro digestion model combined with an in vivo assay. We speculated that the Pb and Cd concentrations, the bioavailability, and the consumption risk would be greatly decreased by the polishing process. The specific objectives were to: (1) determine the Pb and Cd concentrations in rice using different polishing degrees; (2) measure the Pb and Cd bioaccessibility (BAC) in rice based on an in vitro digestion model (PBET); (3) determine the Pb and Cd RBA based on their accumulation in the kidney, liver, and femur tissues of mice fed with Pb-Cd contaminated rice; (4) assess the effect of rice polishing on health risk based on the BAC correction.

## 2. Materials and Methods

### 2.1. Sampling and Rice Polishing

The Pb- and Cd-contaminated rice (*Oryza sativa* L.) was collected from a farmer that lived near the Fankou Pb/Zn Mine in Renhua County, Guangdong Province, China. Four degrees, including brown rice, lightly processed brown rice, germinated rice, and polished rice, were produced successively using an intelligent germinated rice machine (Weide FD-889) with the I and II parallel processing for each degree [17]. The surface morphologies of the rice with the four types of processing precisions were observed using a Nikon SMZ 25-electric fluorescence microscope.

### 2.2. Determination of the Total Pb, Cd, and Mineral Nutrients in Rice

The rice samples with the different processing precisions were dried, ground, and packaged until analysis. The rice sample (approximately 0.3 g) and 5 mL of concentrated high-purity HNO_3_ were digested using a microwave oven digestion system (Multiwave Pro, AntonPaar, Austria). The digested mixture was then diluted with 1% HNO_3_ after being cooled to room temperature. The Pb, Cd, Zn, and Mn concentrations in the rice samples were analyzed using inductively coupled plasma mass spectrometry (ICP-MS, 7700X, Agilent, Santa Clara, CA, USA), and the concentrations of K, Ca, Mg, and Fe were determined using inductively coupled plasma optical emission spectrometry (ICP-OES, Optima 2000DV, PerkinElmer, Waltham, MA, USA). Certified reference material (GBW10045 rice flour) and blanks were determined for quality assurance purposes. The recovery rates for Pb, Cd, K, Ca, Mg, Fe, Mn, and Zn in the standard reference rice were 92.9% ± 2.5%, 93.7% ± 0.8%, 85.1% ± 0.3%, 86.8% ± 12.7%, 91.2% ± 0.4%, 89.5% ± 3.8%, 89.3% ± 0.6%, and 91.23% ± 0.6% (n = 3), respectively.

### 2.3. Evaluation of the In Vitro Bioaccessibility

The physiologically based extraction test (PBET) was adapted to perform the digestive simulations. The detailed procedures can be found in our previous research [18]. The gastric phase was prepared by combining 1.25 g porcine pepsin, 0.5 g malic acid, 0.5 g sodium citrate, 420 μL lactic acid, and 500 μL acetic acid in 1 L ultrapure Milli Q water, and the pH was adjusted to 1.5 ± 0.05 with concentrated HCl. To the intestinal solution was added 5.25 g bile salts and 1.5 g pancreatin in a final volume of 200 mL saturated NaHCO_3_.

Gastric (G) phase: The solutions were sealed and incubated at 37 °C with agitation (150 rpm) for 1 h. Following the incubation, samples were centrifuged, and 5 mL supernatant was filtrated with a 0.45 μm membrane, then the solution was diluted with 1% nitric acid prior to the ICP-MS analysis. Gastrointestinal (GI) phase: Five milliliters of the intestinal solution was added to all the samples, and they were then shaken at 37 °C for 4 h and adjusted to 7.0 ± 0.05 with saturated NaHCO_3_. Following incubation, the sample procedure was the same as utilized in the gastric phase.

The Pb and Cd bioaccessibility in the contaminated rice was measured according to the following equation [8]:

(1) $$\mathrm{Bioaccessibility}\left(\%\right)=\frac{\mathrm{In}\;\mathrm{vitro}\;\mathrm{concentration}}{\mathrm{Total}\;\mathrm{metal}\;\mathrm{concentration}\;\mathrm{before}\;\mathrm{digestion}}\times 100$$

where the in vitro concentration is the soluble concentration in the gastrointestinal phase.

### 2.4. Evaluation of the In Vivo Bioavailability

#### 2.4.1. Mouse Meal Preparation

Brown rice (Pb: 7.14 mg kg^−1^; Cd: 0.82 mg kg^−1^) and polished rice (Pb: 3.02 mg kg^−1^; Cd: 0.71 mg kg^−1^) were selected to measure the Pb and Cd bioavailability in the mouse assay. Uncontaminated rice was collected from public supermarkets to develop the dose–response curve (Supplementary Information). After washing three times, the three types of rice were cooked (2:1 water to rice (weight)) until no water was left. The control rice was contaminated with Pb acetate and CdCl_2_ to obtain the reference rice with 2, 4, 8, and 10 mg kg^−1^ Pb and 0.4, 0.6, 0.8, and 1.2 mg kg^−1^ Cd. All cooked rice samples were then granulated and freeze-dried.

#### 2.4.2. In Vivo Evaluation of the Pb and Cd Exposure

In vivo studies utilized female Balb/C mice that were six weeks of age. All mice were raised in metabolic cages (22 ± 2 °C, 12 h reversed light-dark cycle) and acclimated with water ad libitum for 7 days. The mice were transferred to individual cages (three mice per cage; nine mice per treatment group). After administration of brown rice, polished rice, uncontaminated rice, and Pb acetate-/CdCl_2_-amended rice for 10 d, all mice were weighed and then sacrificed by cervical dislocation and dissected to collect the liver, kidney, and femur tissues (Figure S1). The tissues were stored and freeze-dried for further analysis. The animal work was approved by the Animal Ethics Committee of Guangzhou Huateng Biomedical Technology Co. LTD. The Certificate of Laboratory Animal Ethics is presented in section of Supplementary Information.

A dose–response curve of the metal accumulation in mouse tissues was developed to verify the applicability of the mouse model with Pb acetate-/CdCl_2_-spiked rice administration to the mice (Supplementary Materials and Figure S2). The kidneys, livers, and femurs were chosen to determine the bioavailability of Pb and Cd (Pb RBA/Cd RBA). The RBAs of Pb and Cd were determined according to the following equations [10]:

(2) $$\mathrm{Pb}\;\mathrm{relative}\;\mathrm{bioavailability}\;\left(\%\right)=\frac{{\mathrm{LKF}\;\mathrm{Pb}}_{\mathrm{rice}}}{{\mathrm{LKF}\;\mathrm{Pb}}_{\mathrm{Pb}\;\mathrm{acetate}}}\times \frac{{\mathrm{Pb}\;\mathrm{dose}}_{\mathrm{Pb}\;\mathrm{acetate}}}{{\mathrm{Pb}\;\mathrm{dose}}_{\mathrm{rice}}}\times 100\%$$

(3) $$\mathrm{Cd}\;\mathrm{relative}\;\mathrm{bioavailability}\;\left(\%\right)=\frac{{\mathrm{LKF}\;\mathrm{Cd}}_{\mathrm{rice}}}{{\mathrm{LKF}\;\mathrm{Cd}}_{{\mathrm{CdCl}}_{2}}}\times \frac{{\mathrm{Cd}\;\mathrm{dose}}_{{\mathrm{CdCl}}_{2}}}{{\mathrm{Cd}\;\mathrm{dose}}_{\mathrm{rice}}}\times 100\%$$

where LKF Pb rice and LKF Pb Pb acetate denote the Pb concentration in the livers, kidneys, and femurs of the mice administrated rice and Pb acetate-amended rice, respectively; Pb dose rice and Pb dose Pb acetate denote the dose levels of Pb in the mice administrated rice and Pb acetate-amended rice, respectively; LKF Cd rice and LKF Cd_CdCl2_ refer to the Cd concentrations in the livers, kidneys, and femurs of the mice administrated rice and CdCl_2_-spiked rice, respectively; Cd dose rice and Cd dose _CdCl2_ denote the dose levels of Cd in the originally contaminated rice and CdCl_2_-spiked rice, respectively.

### 2.5. Consumption Risk Assessment

The potential hazardous exposure to Pb and Cd via rice consumption was evaluated based on the estimated daily intake (EDI) and EDI_BAC_ based on the bioaccessibility. The calculation method is as follows:

(4) $$\mathrm{EDI}=\frac{C_{R}\times M}{\mathrm{BW}}$$

(5) $${\mathrm{EDI}}_{\mathrm{BAC}}=\frac{C_{R}\times \mathrm{BAC}\times M}{\mathrm{BW}}$$

where CR is the total Pb or Cd measured in the rice sample (μg kg^−1^); M is the daily rice intake based on the China Health and Nutrition Survey (326 g d^−1^ for the southern population) [19]; BW is the average adult body weight (60 kg for an adult); BAC is the bioaccessibility of Pb and Cd in the contaminated rice.

The target hazard quotient (HQ) for Pb and Cd based on the total or bioavailability data was calculated using the following equation:

(6) $$\mathrm{HQ}=\frac{\mathrm{EF}\times \mathrm{ED}\times C\times M}{\mathrm{RfD}\times \mathrm{AT}\times \mathrm{BW}}\times 10^{-3}$$

where C denotes the soluble concentration of metal during digestion; M is the daily consumption rate (g person^−1^ day^−1^); BW denotes the average adult body weight (60 kg); ED is the exposure duration (70 years); EF denotes the exposure frequency (365 days per year); AT represents the average time for noncarcinogens (ED×EF); RfD denotes the oral reference dose of Pb (3.57μg kg^−1^ day^−1^) and Cd (0.83 μg kg^−1^ day^−1^) [20]; 10^−3^ is the unit conversion factor.

### 2.6. Quality Control and Statistical Analyses

All concentrations in the samples, metal bioaccessibility and RBA, and HQ data are shown as the means ± standard deviations of the three repeats. An independent-sample t-test was adopted for the Pb and Cd RBA. Differences were conducted using a variance analysis followed by Tukey’s multiple comparisons with a significance level of *p* < 0.05. All statistical analyses were performed using SPSS 23.0 (IBM, Armonk, NY, USA)

## 3. Results

### 3.1. Effect of the Processing Precision on the Surface Morphology of Rice

**Figure 1:**
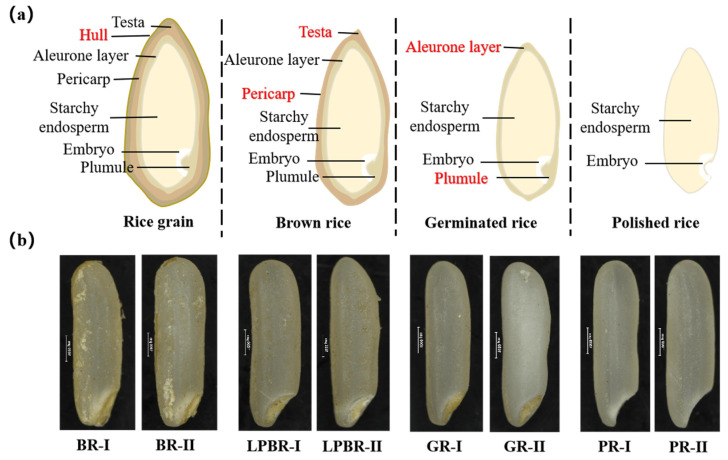
(**a**) Internal structure of mature rice grain and rice with different polishing degrees; (**b**) surface morphology of rice with four degrees of polishing. BR, brown rice; LPBR, lightly processed brown rice; GR, germinated rice; PR, polished rice. Ⅰ and Ⅱ indicate the two parallel processing for each polishing degree. The scale bar shown in the Figure 1b is 1000 µm.

The internal structures of a mature rice grain and rice with different polishing degrees are shown in Figure 1a. A rice grain is composed of a husk and a fruit, and the fruit obtained after removing the husk is called brown rice [21]. Brown rice consists of the endosperm and rice bran layers, which are composed of a testa, a pericarp, an aleurone layer, and an embryo (scutellum, epiblast, plumule, and radicle) [22]. The polished rice for daily consumption is the starchy endosperm and a small amount of the aleurone layer and embryo, while germinated rice is polished rice that retains a portion of the plumule.

In this study, the surface morphology of brown rice, light brown rice, germinated rice, and polished rice was observed using a Nikon SMZ25-motorized body fluorescence microscope, as shown in Figure 1b. The brown rice retained a relatively complete embryo and bran layer, and the color of the rice was deeper and still had the brownish outer bran layer. At this stage of the germinated rice, the surface of the rice was mechanically damaged, and the surface was uneven, but still retained a relatively complete plumule. The color of the polished rice was remarkably lighter than that of the brown rice. The results of this study support the evidence from previous observations [17]. The extent of removal of the bran layer on the surface of the polished kernels depends on the degree of polishing, and thus, it is related to the whiteness of the grain. Overall, these results indicated that the processing precision had significantly changed the surface morphology of the rice and the degree of the residual aleurone layer, the testa, and the pericarp on the surface of the rice.

### 3.2. Total Concentrations of Pb, Cd, and Nutritional Elements in Rice

**Figure 2:**
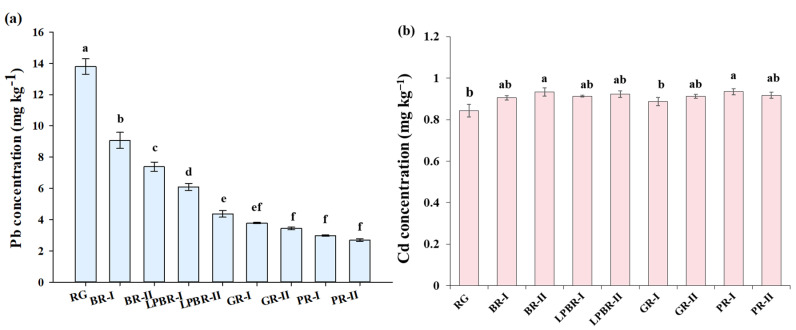
Concentrations of Pb (**a**) and Cd (**b**) in rice with four types of polishing degrees. RG, rice grain; BR, brown rice; LPBR, lightly processed brown rice; GR, germinated rice; PR, polished rice. Ⅰ and Ⅱ indicate the two parallel processing in each polishing degree. Different lowercase letters indicate that the concentrations of lead and cadmium in rice with different polishing degrees differ significantly at *p* < 0.05.

The concentrations of Pb and Cd in the rice with different polishing degrees are shown in Figure 2. The Pb concentration in the original rice grain was 13.76 mg kg^−1^. The Pb concentration in the brown rice was significantly higher than that in the light brown rice, germinated rice, and polished rice (*p* < 0.05). With an increase in the polishing degree, the Pb removal rates were 40%, 62%, 74%, and 79% in the brown rice, light brown rice, germinated rice, and polished rice, respectively. However, no significant difference was found between the Cd concentration in the four types of rice with the four polishing degrees.

The impact of rice polishing on the total amount of Pb and Cd in the rice grains was different due to the differences in their distribution and combination in the rice grains. Approximately 36.03% ± 1.15% Pb and 9.40% ± 1.46% Cd were removed by processing brown rice into polished rice [23]. This was supported by observations that the element Pb had the most uneven distribution in the rice, and the removal rates of Pb and Cd were 41.40% and 20.71% by polishing, respectively [24]. This could be related to the fact that the Pb distribution in the rice displayed a high heterogeneity, and more Pb may have been distributed in the chaff layer. Conversely, different proportions of Cd concentrations in the embryo (40%), endosperm (45%), and chaff (15%) were found by Liu et al. [25], although the dry weight of the embryo was only an average 9% of the grain. The results of this study found that there was a slight decline in the Cd concentration of rice with the different processing precisions, and this may have been related to the large differences in the Cd concentration in the different parts of the different rice cultivars. A similar result was shown by Huang et al. [15], who found that a significant decline was observed in the As, Pb, and Cr concentrations in polished rice compared with those in brown rice, while the Cd content did not change much. In this study, with the removal of rice husks and a portion of the bran layers, there was no decreasing trend in the Cd concentration of the rice with various processing precisions, indicating that Cd was rarely present in the husk and bran layers. It is present primarily in the endosperm and binds to rice protein [26]. Hence, it is difficult to remove using rice polishing.

The rice polishing process also had an influence on the concentrations of the nutritional elements in rice. The results showed that the concentrations of K, Ca, Fe, Mg, Mn, and Zn were strikingly different between the different processing precisions (*p* < 0.05) (Table 1). The concentration of K was the highest in the rice, with the Mn element being the lowest. With improvement in the rice polishing precision, the mineral element concentrations showed a decreasing trend, and the element loss rate of germ rice I was the highest. The K, Mg, and Mn concentrations of the polished rice had little differences compared with the brown rice, while the concentrations of Ca, Fe, and Zn were reduced by approximately 14%, 25%, and 70%, respectively.

The loss rate of the six elements caused by the same processing varied with the elements, and this was due to the uneven distribution of the elements in the rice and variations among the cultivars. The decreasing trend in the brown rice was in accordance with the previous studies. Mn, Fe, P, and K were primarily located in the outer layer of the grain, and Cd and Zn were accumulated in both the endosperm and bran [27]. Thereby, different amounts of toxic metal and nutrient elements were removed by polishing treatment [28].

**Figure 3:**
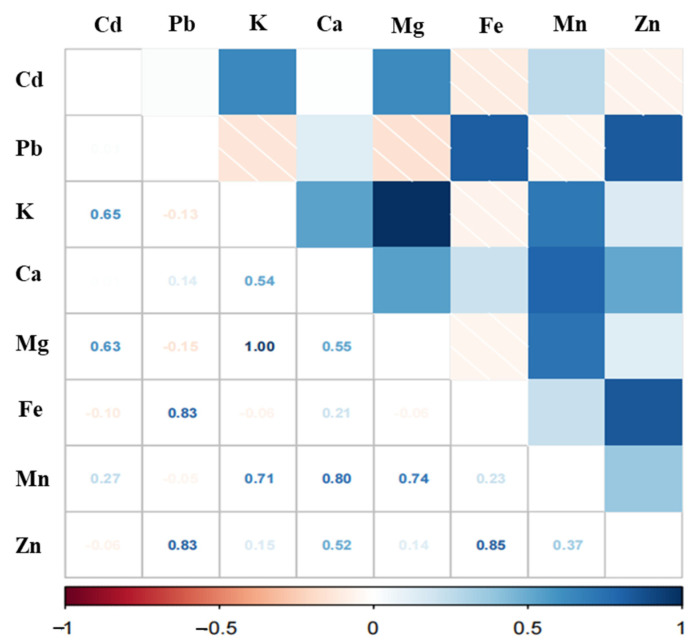
The correlation between elements in rice with different polishing degrees.

The correlation analysis results of the elements in the rice with different processing precisions is shown in Figure 3. A perfect positive correlation was found between K and Mg (r = 1.00, *p* < 0.05). Meanwhile, significant positive correlations existed between Fe and Pb (r = 0.83, *p* < 0.05), Zn and Pb (r = 0.83, *p* < 0.05), K and Cd (r = 0.65, *p* < 0.05), and Mg and Cd (r = 0.63, *p* < 0.05). This also verified that the removal rates of Pb, Fe, and Zn increased simultaneously, while the concentrations of Cd, Mg, and K did not change greatly with the deepening of the processing precision (Table 1). A synergistic absorption phenomenon between the Pb, Fe, and Zn concentrations was found, and the mineral element concentrations in the rice samples were affected by factors such as the varieties and cultivation measures. This is in agreement with earlier reports by Jiang et al. [29]. The correlation between elements in the rice may depend on the ecological environment, plant absorption of the different active state elements, and the operation of the elements. Hence, the reasons for the synergy or antagonism between elements are complex and require further investigation.

### 3.3. Effect of Polishing on the Bioaccessibility of Pb and Cd in Rice

**Figure 4:**
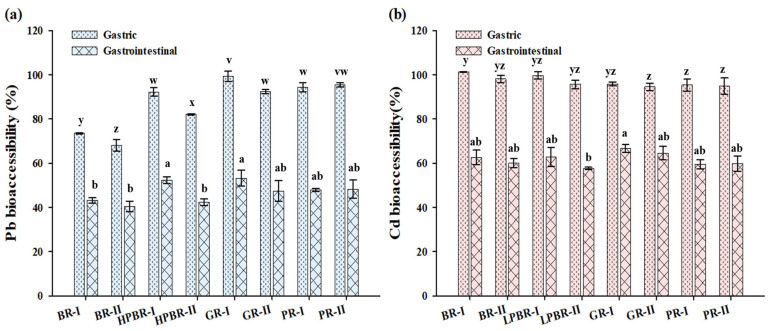
Effects of different polishing degrees on the bioaccessibility of Pb (**a**) and Cd (**b**) in rice. BR, brown rice; LPBR, lightly processed brown rice; GR, germinated rice; PR, polished rice. Ⅰ and Ⅱ indicate the two parallel processing in each polishing degree. xyz and abc indicate that the bioaccessibility of Cd in the gastric and intestinal phases in rice with different polishing degrees differs significantly at *p* < 0.05.

The BAC of Pb and Cd in the four types of processed rice determined by the gastric and gastrointestinal fractions are shown in Figure 4. There were significant differences between the Pb BAC (the average value of parallel processes I and II for each type) in the rice with different polishing precisions (*p* < 0.05). In the gastric fraction, the Pb BAC followed the order of germinated rice (99%) > polished rice (94%) > lightly processed brown rice (87%) > brown rice (70%). The same declining trend was found in the gastrointestinal fraction. The Cd BACs in the gastric fraction were 104%, 98%, 95%, and 95% for brown rice, lightly processed brown rice, germinated rice, and polished rice, respectively. From the present study, more soluble Cd released from the gastric fractions compared with the alkalic gastrointestinal fractions, which is consistent with a previous finding using the PBET method [18].

A possible explanation for the high Pb and Cd BAC in the germinated rice is the high solubility of Pb and Cd in the embryo, and the concentrations of Cd and Pb in the embryo and plumule were higher than those in the endosperm [30]. Peng et al. [31] reported that the Cd BAC in cooked rice was less than 50%, but it increased with the increase of the polishing degree. In this study, there was no pattern between the Cd BAC and the polishing degree. This may have been related to the properties of the rice and the selection of the in vitro simulated gastrointestinal digestion with the influence of the pH, the enzyme composition, the solid–fluid ratio, and the digestion time on the element bioaccessibility.

### 3.4. Effect of Polishing on the Bioavailability of Pb and Cd in Rice

After 10 days of feeding mice Pb-Cd-contaminated rice with different polishing precisions, the Pb and Cd concentrations in the livers, kidneys, and femurs of the mice were determined (Figure S3). The Pb and Cd RBA in the polished rice and brown rice based on the tissue Pb and Cd accumulations varied considerably (Table 2). The Pb RBA in the livers, kidneys, and femurs of the mice in the polished rice group was 26.6% ± 1.68%, 65.3% ± 0.83%, and 19.0% ± 1.39%, which was 1.6-, 2.6-, and 1.5-times higher than that in the brown rice group, respectively. The Cd RBA in the livers (44.1%) and kidneys (52.1%) in the polished rice group were higher than that in the brown rice group (33.2% for the livers and 38.8% for the kidneys). Similar observation by Liu et al. [32] showed that, after feeding female rats for 24 h with contaminated brown and polished rice, the absorption rates of Cd in the gastrointestinal tracts were found to be 10% and 15%, respectively. This is likely due to the fact that dietary fiber, vitamins, minerals, lipids, and proteins in the outer layer of rice enter the gastrointestinal tract together, and subsequently, these dietary components may affect the absorption and toxicity of heavy metals during co-digestion [33]. Studies have reported that an increased intake of bioavailable zinc and iron in fishermen who eat zinc- and iron-rich oysters reduces the excessive absorption of cadmium [34]. Studies in rats exposed to dietary lead have shown a strong correlation between increased intestinal lead absorption and iron deficiency in the body [35]. Previous studies have shown that dietary fiber (such as wheat bran) may bind to metals and decrease their absorption, while minerals such as Fe, Zn, Mg, and Ca in hulls or the aleurone may also inhibit the absorption of heavy metals by competing or downregulating for the linkage to divalent metal transporters (DMT1) or binding to metallothionein (MT-I and MT-II), and these processes increase intestinal absorption [36,37,38].

**Figure 5:**
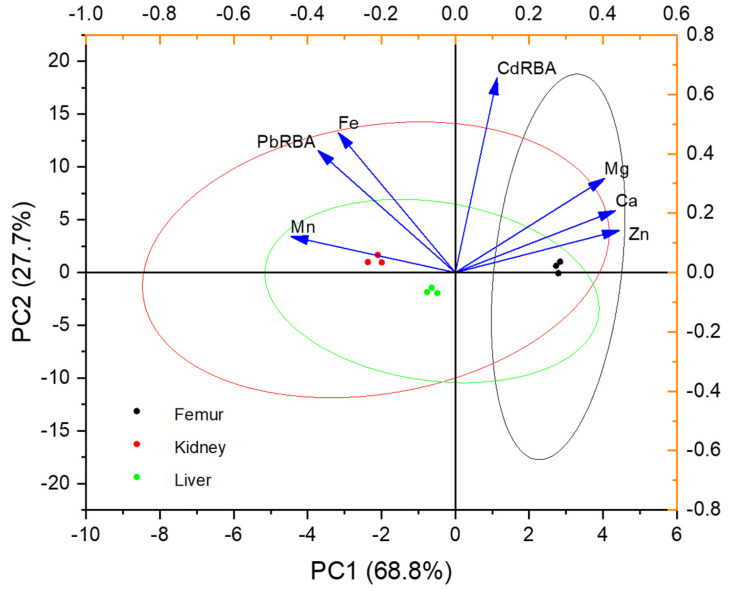
Principal component analysis of various mineral elements and metal relative bioavailability in different mouse organs.

Lead and Cd are non-essential elements in the body and may interact with other elements synergistically or in antagonism once ingested in the body. In this study, principal component analysis (PCA) was carried out based on the Pb/Cd RBA and minerals in the mouse organs. The first component (PC1) can be interpreted as minerals related to the absorption of Cd in the mouse organs. The second component (PC2) can be interpreted as minerals that are related to the Pb RBA. The present results showed that the Pb RBA was positively correlated with Fe and Mn (Figure 5), and the Cd RBA was positively correlated with Ca, Mg, and Zn in the kidneys and livers. Similarly, the direct effect of lead intake on the increase of Fe or Mn was found in the organs of rats [39], which was attributed to Mn and Fe can be transported via the same molecular mechanisms [40]. A similar result was reported by Alonso et al. [41], who observed that Cd accumulation in cattle tissues was positively correlated with Ca, Co, and Zn in the kidney and liver.

In this study, Cd absorption might be also affected by Pb, and the co-exposure to Cd and Pb reduced the Pb RBA in the organs, possibly due to the interaction of other essential metal elements in the rice matrix (e.g., Ca, Mg, Fe, Mn, and Zn) and competitive uptake through divalent metal transporters [42]. Since Pb and Cd are non-essential elements in the body, there are no specific pathways and carriers specifically responsible for Cd absorption and transport. Therefore, their transport and accumulation in an organ are primarily due to the overexpression of transporters or changes in metal segmentation. The overexpression of transporters may be the result of an attempt by the divalent metal transporter (DMT1) to adapt to reduced Fe, which is replaced by Cd [43,44]. DMT1 is a nonspecific metal transporter capable of transporting many metals [45]. Interactions between Pb and the matrix may affect the dissolution of Pb under gastric conditions so that the availability of Pb for absorption in the digestion process is restricted [46,47]. In the present study, the concentrations of Pb and Cd accumulation in the livers and kidneys of the brown rice group were lower than that in the polished rice group, which may have been related to the high concentration of Fe in the brown rice. The absorption of Pb through the gastrointestinal barrier is inhibited due to the higher amount of soluble Fe occupying the divalent metal transporter [48]. Hence, the Pb accumulation in the livers and kidneys would decrease. A significant negative correlation between Fe and the Pb RBA or Cd RBA in the kidneys was also found in an analysis of the relationship between the Pb or Cd RBA and the nutrient elements.

### 3.5. Health Risk Assessment

The EDIs of Pb and Cd based on daily rice consumption are presented in Table 3. The Pb EDI calculated by the total amount was 1.9–2.5-times that of the Pb EDI_BAC_, and the Cd EDI was 1.5–1.7-times that of the Cd EDI_BAC_. The above results indicate that calculating the intake in terms of the total heavy metals may overestimate the health risk of rice. The highest Pb EDI_BAC_ was found in brown rice, followed by lightly processed brown rice, germinated rice, and polished rice. There was no obvious change in the Cd EDI_BAC_ between the rice with different polishing degrees. The Pb and Cd HQs obtained via rice consumption with the four different polishing degrees for adults are shown in Figure S4. With an increase in the polishing degree, the total amount of Pb showed a downward trend (see Figure 2), and rice polishing significantly reduced the total Pb HQ: brown rice (5.27) > lightly processed brown rice (3.85) > germinated rice (2.78) > polished rice (2.08). The Pb HQs of the above polished rice were greater than one, which means that long-term rice consumption will pose potential harm and risk to the health of the consumer.

It is noteworthy that the bioavailability-corrected HQ of Pb (1.36) and Cd (1.99) in the polished rice was higher than those in the brown rice, which deserves serious attention (Table 2). Although the total amount of Pb in polished rice decreases linearly after processing, it cannot offset the increase of HQ due to the Pb RBA in the polished rice being significantly higher than the Pb RBA in brown rice. The bioavailability-corrected HQ of Cd in rice also elevated with the increase of the Cd RBA in the polished rice compared with that in the brown rice. The present result reveals that local residents are facing serious food safety problems whether they eat brown rice or polished rice. Consequently, some effective measures may be necessary to resolve heavy metal contamination in soil or prohibit planting in and supplying rice from this area to ensure the health of the people. For populations for whom it is hard to avoid contact with contaminated rice, another choice would be to avoid the risks by consuming more mineral elements in their daily diet, diversifying their diet structure, and maintaining balanced nutrition.

## 4. Conclusions

Our research provides important information about rice polishing and risk control. Rice polishing may reduce the total concentration of heavy metals in polished rice, but it may not reduce the health risk through rice consumption. Although rice polishing remarkably reduces a large proportion of the total Pb in polished rice, the Pb HQ based on bioavailability was equal to that of the brown rice group. It is noteworthy that the Cd HQ in polished rice was higher than that in brown rice, despite no difference being found in the total Cd between the two types of rice. Thus, in order to minimize the Pb and Cd exposure related to rice consumption, further research is needed to reduce the metal bioavailability in rice during digestion.

## Figures and Tables

**Table 1:** Concentrations of mineral elements in rice with different polishing degrees (mg kg^−1^, dw, Mean ± SD, *n* = 3).

Polishing Precision	Nutritional Elements					
	K	Ca	Mg	Fe	Mn	Zn
BR-Ⅰ	2501 ± 12.8 d	125 ± 5.1 a	856 ± 4.3 d	366 ± 20.6 a	25 ± 0.3 b	48 ± 1.4 a
BR-Ⅱ	2451 ± 31.7 d	103 ± 2.2 b	843 ± 16.9 d	255 ± 22.2 c	23 ± 0.6 d	39 ± 1.1 c
LPBR-Ⅰ	2449 ± 9.6 d	104 ± 0.5 b	844 ± 3.4 d	324 ± 12.8 a	23 ± 0.1 cd	42 ± 0.6 b
LPBR-Ⅱ	2735 ± 20.5 a	119 ± 1.4 a	976 ± 6.7 a	309 ± 33.1 b	26 ± 0.4 a	41 ± 0.1 bc
GR-Ⅰ	1914 ± 11.4 e	104 ± 10.0 b	616 ± 6.4 e	191 ± 2.2 d	22 ± 0.2 d	36 ± 0.4 d
GR-Ⅱ	2658 ± 18.3 b	115 ± 1.3 ab	943 ±7.0 b	187 ± 4.9 d	24 ± 0.1 c	38 ± 0.1 cd
PR-Ⅰ	2770 ± 34.9 a	120 ± 0.3 a	988 ± 5.8 a	120 ± 0.5 e	25 ± 0.1 b	38 ± 0.2 cd
PR-Ⅱ	2587 ± 25.4 c	107 ± 1.6 b	884 ± 7.8 c	109 ± 0.1 e	23 ± 0.3 cd	36 ± 0.5 d

Note: BR, brown rice; LPBR, lightly processed brown rice; GR, germinated rice; PR, polished rice. Ⅰ and Ⅱ indicate the two parallel processing in each polishing degree. Different lowercase letters in the same column indicate significant differences in the concentration of the same element in rice, with different polishing degrees at *p* < 0.05.

**Table 2:** Pb and Cd relative bioavailability (RBA) and bioavailability-corrected hazard quotient (HQ) in mouse organs by consuming brown rice or polished rice (%, Mean ± SD, *n* = 3).

Polishing Degrees	Pb RBA			HQ ^a^	Cd RBA		HQ ^a^
	Liver	Kidneys	Femur		Liver	Kidneys	
Brown rice	16.5 ± 1.29b	25.3 ± 3.56b	12.3 ± 1.33b	1.33	33.2 ± 9.55b	38.8 ± 3.86b	1.44
Polished rice	26.6 ± 1.68a	65.3 ± 0.83a	19.0 ± 1.39a	1.36	44.1 ± 3.09a	52.1 ± 5.76a	1.90

Note: Different lowercase letters in the same column indicate that Pb RBA and Cd RBA in mouse organs differ significantly at *p* < 0.05 between the brown rice and polished rice groups. HQ ^a^ was corrected by Pb or Cd bioavailability in the mouse kidney.

**Table 3:** Estimated daily intake (EDI, µg kg^−1^ BW day^−1^) of based on total amount and bioaccessibility of Pb and Cd by consuming rice with different polishing degrees.

Processing Precision	Pb		Cd	
	EDI_total_	EDI_BAC_	EDI_total_	EDI_BAC_
BR-Ⅰ	49.38	21.32	4.95	3.11
BR-Ⅱ	40.23	16.27	5.06	3.04
LPBR-Ⅰ	33.21	17.40	4.95	3.11
LPBR-Ⅱ	23.79	10.07	5.01	2.89
GR-Ⅰ	20.58	10.96	4.85	3.23
GR-Ⅱ	18.67	8.87	4.95	3.20
PR-Ⅰ	16.22	7.77	5.12	3.05
PR-Ⅱ	14.64	7.06	5.01	3.00

Note: BR, brown rice; LPBR, light processed brown rice; GR, germinated rice; PR, polished rice. Ⅰ and Ⅱ indicate two parallel processing in each polishing degree.

## Data Availability

The datasets generated to obtain the results presented in this article are available from the corresponding authors upon reasonable request (hydu@mail.hzau.edu.cn).

## Supplementary Materials

The following Supporting Information can be downloaded at: https://www.mdpi.com/article/10.3390/foods11172718/s1, Figure S1. Flow chart for the model of the exposure of mice to mixed food; Figure S2. Dose–response curve for Pb and Cd accumulation in the kidneys (●), liver (■), and femur (▼); Figure S3. Effects of rice with different polishing degrees on the concentrations of Pb and Cd in mouse organs. (a) and (b) indicate Pb and Cd, respectively. xyz, abc, and ABC, respectively, show that the concentrations of Pb and Cd in the liver, kidneys, and femur of mice after feeding them rice with different polishing degrees differ significantly at *p* < 0.05.; Figure S4. The hazard quotient (HQ) of the total Pb and Cd by consuming rice with different polishing degrees. (a) and (b) indicate Pb and Cd, respectively. BR, brown rice; LPBR, lightly processed brown rice; GR, germinated rice; PR, polished rice. Ⅰ and Ⅱ indicate the two parallel processing in each polishing degree. Different lowercase letters indicate that the HQ of Pb by consuming rice with different polishing degrees differs significantly at *p* < 0.05.
