# Supplementary material for: Effect of Polishing on Lead and Cadmium Bioavailability in Rice and Its Health Implications
Source: Foods. 2022 Sep 5;11(17):2718. doi: 10.3390/foods11172718 (PMC9455439; doi:10.3390/foods11172718)
Supplement: Supplementary file 1 [file foods-11-02718-s001.zip › foods-1846101-supplementary.pdf]

Supplementary material

Effect of Polishing on Lead and Cadmium Bioavailability in Rice and Its Health Implications

Xianghua Chen [1,2,3], Fangman Chen [1,3], Shuo Sun [1,3], Yingwen Li [1], Yongxing Li [1], Hui Mo [1], Zhian Li [1,2] and Ping Zhuang [1,2,*]

[1] South China Botanical Garden, Chinese Academy of Sciences, Guangzhou 510650, China
[2] Southern Marine Science and Engineering Guangdong Laboratory (Guangzhou), Guangzhou 511458, China
[3] University of Chinese Academy of Sciences, Beijing 100049, China
* Correspondence: zhuangp@scbg.ac.cn

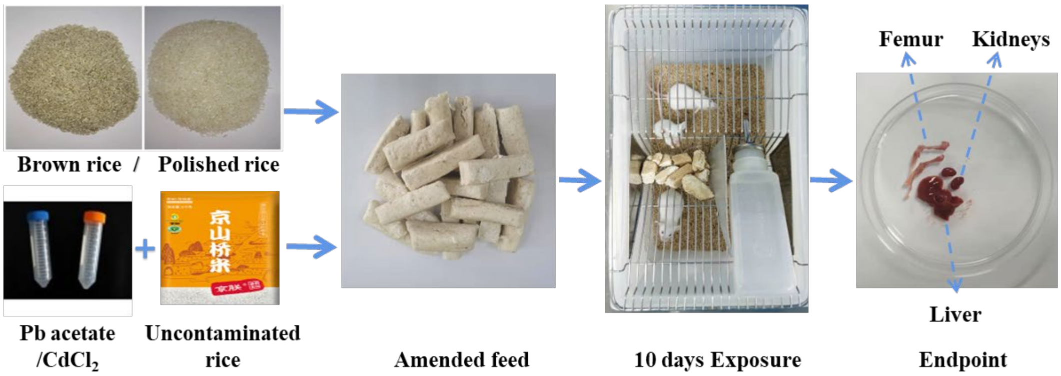

Figure S1. Flow chart for exposure of mixed food to mouse model.

Material and methods

Dose-response curve

Before determining Pb and Cd RBA in rice, a dose-response relationship was established based on a mouse assay following exposure (10 days) of four dose levels of Pb acetate-spiked rice (2, 4, 6 and 8 mg kg^{-1}) and $CdCl_2$-spiked rice (0.4, 0.6, 0.8 and 1 mg kg^{-1}) to mice and quantification of Pb and Cd concentrations in kidneys, liver, and femur (Fig.7). Strong linear relationships (R^2 = 0.87-0.95) were found among Pb accumulation in the kidneys, liver, or femur and Pb doses level (286-1335 μg Pb kg^{-1} d^{-1}), suggesting the suitability of using these endpoints to calculate Pb RBA in rice. Both kidneys and femur are target organs that can be used to determine Pb RBA in rice, and the concentration of Pb in kidneys > femur > liver. Similar linear dose responses have been observed for Pb accumulation in the liver, kidneys, and bone in a swine model (Casteel et al., 2006). There is a strong linear relationship between Cd accumulation in mouse kidneys (R^2 = 0.96) and liver (R^2 = 0.95) and cadmium exposure doses (58-136 μg Cd kg^{-1} d^{-1}), both of kidneys and liver can be used to determine Cd RBA in rice.

Previous studies have used a single gavage dose and area under mouse blood Pb concentration time curve (AUC) as the endpoint to determine Pb RBA under fasted state (Juhasz et al., 2014). The optimal fit of the linear model comes from bone or blood Pb (R^2 = 0.96), and the stability of Pb RBA in femur may reflect its role as the primary source of Pb redistribution between tissues. Compared to the mouse blood AUC model, steady stated dose approach provided better repeatability of using Pb and Cd accumulation in the tissues as the endpoint and the advantage of using much fewer animals (Li et al., 2017).

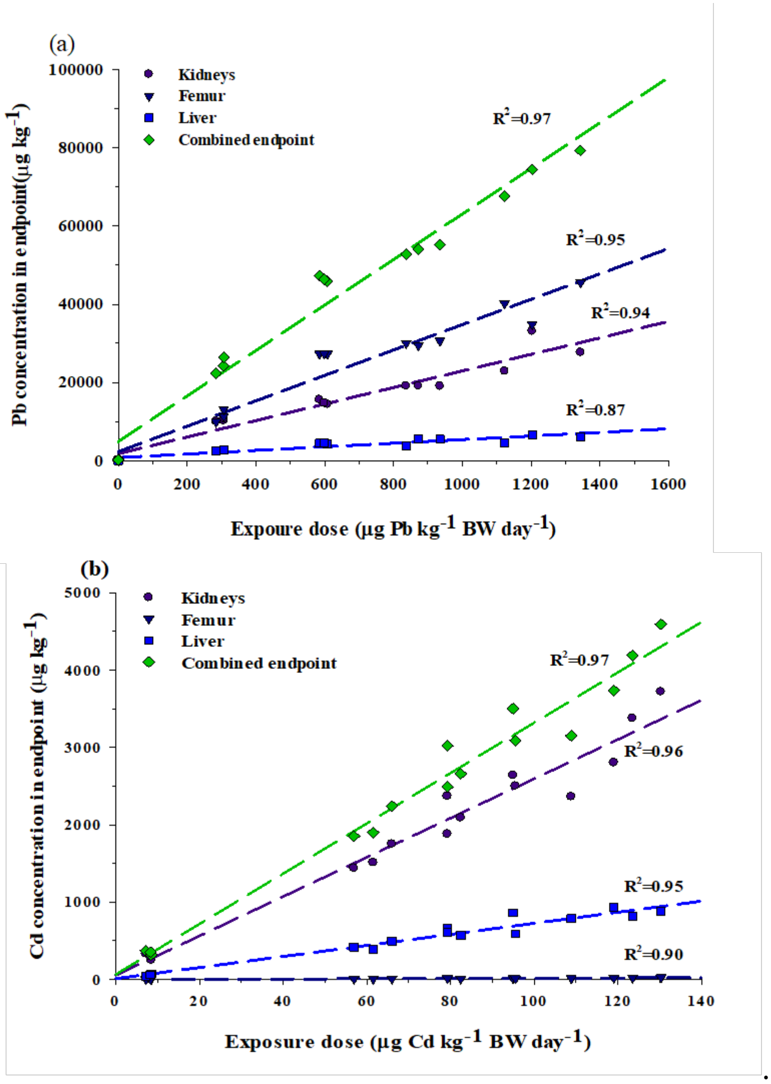

Figure S2. Dose response curve for Pb and Cd accumulation in the kidneys (●), liver (■), femur (▼) and combined endpoint (◆) of mice dosed with Pb acetate/CdCl2-amended rice over a 10 days exposure period.

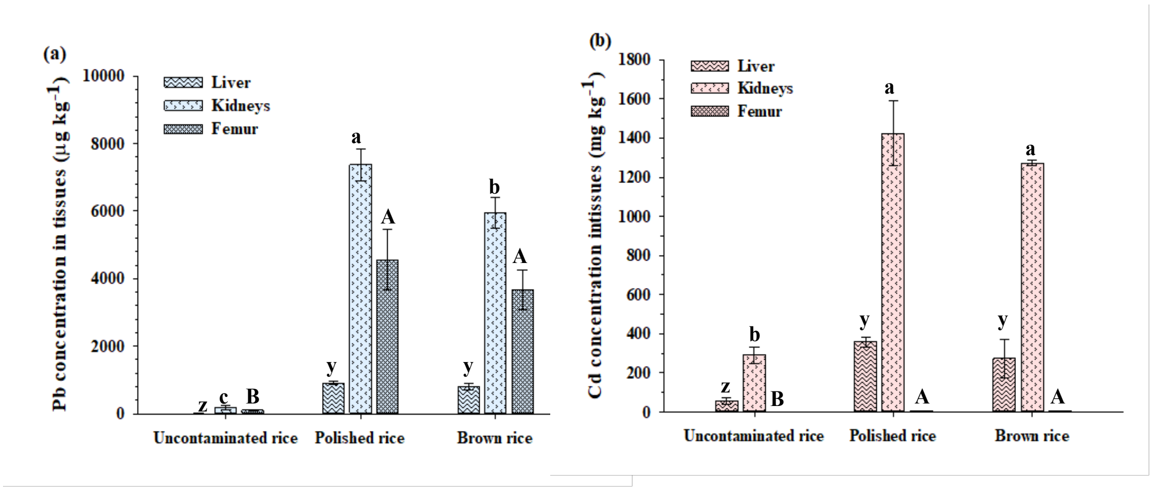

Figure S3. Effects of rice with different milling degrees on the concentrations of Pb and Cd in mouse organs. (a) and (b) indicate Pb and Cd, respectively. xyz, abc, and ABC, respectively, show that the concentrations of Pb and Cd in liver, kidneys, and femur of mouse after feeding rice with different milling degrees differ significantly at $p < 0.05$.

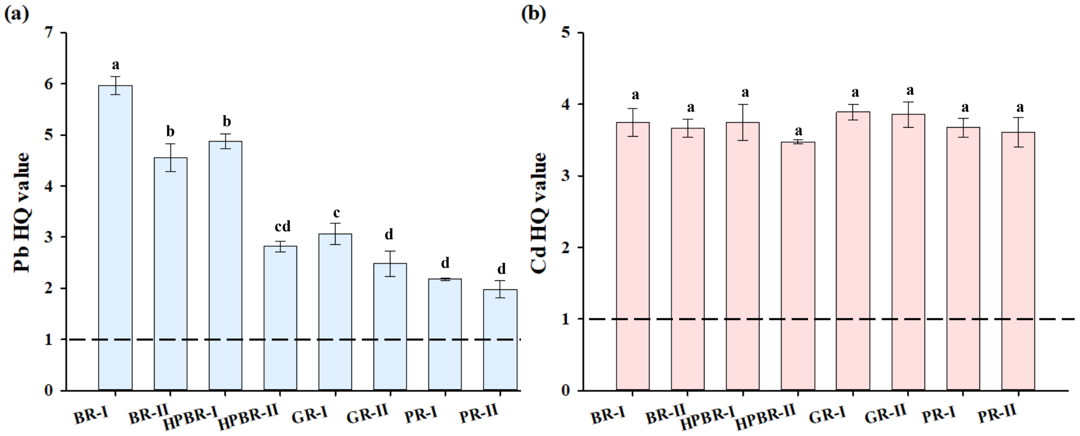

Figure S4. The target hazard quotient (HQ) of total Pb and Cd by consuming rice with different milling degrees. (a) and (b) indicate Pb and Cd, respectively. BR, brown rice; LPBR, light processed brown rice; GR, germinated rice; PR, polished rice. I and II indicate two parallels in each milling degree. Different lowercase letters indicate that the HQ of Pb by consuming rice with different milling degrees differs significantly at p <0.05.

References

1. Casteel, S.W., Weis, C.P., Henningsen, G.M., Brattin, W.J. (2006) Estimation of relative bioavailability of lead in soil and soil-like materials using young swine. Environmental Health Perspectives 114, 1162-1171.
2. Juhasz, A.L., Gancarz, D., Herde, C., McClure, S., Scheckel, K.G., Smith, E. (2014) In situ formation of pyromorphite is not required for the reduction of in vivo Pb relative bioavailability in contaminated soils. Environmental Science & Technology 48, 7002-7009.
3. Li, S.W., Sun, H.J., Wang, G., Cui, X.Y., Juhasz, A.L., Li, H.B., Ma, L.Q. (2017) Lead relative bioavailability in soils based on different endpoints of a mouse model. Journal of Hazardous Materials 326, 94-100.
